# Prognostic implication of myocardial perfusion and contractile reserve in end-stage renal disease: A direct comparison of myocardial perfusion scintigraphy and dobutamine stress echocardiography

**DOI:** 10.1007/s12350-021-02844-y

**Published:** 2021-11-08

**Authors:** Joachim Bautz, Jörg Stypmann, Stefanie Reiermann, Hermann-Joseph Pavenstädt, Barbara Suwelack, Lars Stegger, Kambiz Rahbar, Stefan Reuter, Michael Schäfers

**Affiliations:** 1 Department of Nuclear Medicine, University Hospital Münster, Albert-Schweitzer-Campus 1, Building A1, 48149 Münster, Germany; 2 Department of Cardiology, University Hospital Münster, Münster, Germany; 3 Department of Internal Medicine D, Nephrology, University Hospital Münster, Münster, Germany; 4 European Institute for Molecular Imaging, University of Münster, Münster, Germany; 5 DFG EXC 1003 ‘Cells in Motion’ Cluster of Excellence, University of Münster, Münster, Germany

**Keywords:** SPECT myocardial perfusion scintigraphy, Dobutamine stress echocardiography, coronary angiography, chronic kidney disease, end-stage renal disease, coronary artery disease

## Abstract

**Background:**

We aimed to compare the prognostic value of myocardial perfusion scintigraphy (MPS) and dobutamine stress echocardiography (DSE) in patients with end-stage renal disease (ESRD) without known coronary artery disease.

**Methods:**

Two-hundred twenty-nine ESRD patients who applied for kidney transplantation at our centre were prospectively evaluated by MPS and DSE. The primary endpoint was a composite of myocardial infarction (MI) or all-cause mortality. The secondary endpoint included MI or coronary revascularization (CR) not triggered by MPS or DSE at baseline.

**Results:**

MPS detected reversible ischemia in 31 patients (13.5%) and fixed perfusion defects in 13 (5.7%) patients. DSE discovered stress-induced wall motion abnormalities (WMAs) in 28 (12.2%) and at rest in 18 (7.9%) patients. MPS and DSE results agreed in 85.6% regarding reversible defects (κ = 0.358; *P* < .001) and in 90.8% regarding fixed defects (κ = 0.275; *P* < .001). Coronary angiography detected relevant stenosis > 50% in only 15 of 38 patients (39.5%) with pathological findings in MPS and/or DSE. At a median follow-up of 8 years and 10 months, the primary endpoint occurred in 70 patients (30.6%) and the secondary endpoint in 24 patients (10.5%). The adjusted Cox hazard ratios (HRs) for the primary endpoint were 1.77 (95% CI 1.02-3.08; *P* = .043) for perfusion defects in MPS and 1.36 (95% CI 0.78-2.37; *P* = ns) for WMA in DSE. The secondary endpoint was significantly correlated with the findings of both modalities, MPS (HR 3.21; 95% CI 1.35-7.61; *P* = .008) and DSE (HR 2.67; 95% CI 1.15-6.20; *P* = .022).

**Conclusion:**

Perfusion defects in MPS are a stronger determinant of all-cause mortality, MI and the need for future CR compared with WMAs in DSE. Given the complementary functional information provided by MPS vs DSE, results are sometimes contradictory, which may indicate differences in the underlying pathophysiology.

**Supplementary Information:**

The online version contains supplementary material available at 10.1007/s12350-021-02844-y.

## Introduction

Chronic kidney disease (CKD) is a severe global health problem^1^ with a prevalence of approximately 13% of the adult United States population.^2^ It is generally accepted that a reduced glomerular filtration rate (GFR) and albuminuria are independent risk factors for cardiovascular disease and mortality.^3^ Importantly, the risk of cardiovascular events increases with declining kidney function, making it more likely to die from a cardiovascular event than to develop end-stage renal disease, ESRD.^4,5^ Traditional risk factors for coronary artery disease (CAD) such as diabetes mellitus, hypertension, dyslipidaemia, or smoking are frequently present in CKD patients but seem of have less incremental contribution given the underlying severity of their vasculopathy.^6^ It was suggested that chronic inflammation, Ca^+^/PO_4_^2^ regulation disorders, and retention of uremic toxins lead to cardiovascular remodelling different from common atherosclerosis.^7–9^ Accordingly, European Guidelines recommend to classify the cardiovascular event risk for patients with a GFR < 30 mL·min^−1^·1.73 m^−2^ as very high, regardless of the presence or absence of traditional risk factors.^10^ This underlines the importance for cardiovascular screening before kidney transplantation (KTx). In this regard, a non-invasive cardiac stress testing is recommended by the current guidelines.^11,12^ However, the choice of the imaging method is left to the attending physician, primarily because performance and comparability in ESRD cohorts have not been sufficiently explored. MPS and DSE are widely available and affordable imaging modalities that also proved to have a high prognostic impact in cardiovascular risk stratification.^13–16^ Coronary angiography (CA) is currently the reference standard for diagnosing epicardial coronary stenoses.^17^

The aim of this study is to investigate and compare the prognostic value of MPS and DSE in ESRD patients without known CAD who apply for KTx.

## Materials and Methods

### Study Design

We prospectively recruited ESRD patients applying for KTx between January 2010 and June 2012 in our centre. Criteria for inclusion were GFR < 20 mL·min^−1^·1.73 m^−2^ or need for dialysis and age ≥ 18 years. A systematic analysis of cardiovascular risk factors was performed using structured interviews with a physician, health records, and blood lipid levels. In 230 patients without known ischemic heart disease or typical angina pectoris, MPS and DSE were performed as routine clinical procedures, both within one month. There was no predetermined sequence of tests. One patient was unavailable immediately after imaging so the final study cohort consisted of 229 patients. Patients with evidence of ischemia in MPS and/or DSE were evaluated for CA on clinical grounds. At time of imaging, there were 201 patients on dialysis; the remaining 28 patients were prepared for upcoming dialysis.

Patients were followed until October 1, 2020, or death. The primary end-point was defined by a composite of nonfatal myocardial infarction (MI) or all-cause death. The secondary end-point included nonfatal and fatal MI or coronary revascularization (CR) by percutaneous coronary intervention (PCI) or coronary artery bypass grafting (CABG) not triggered by baseline MPS or DSE. Outcome data was collected from medical charts and contacting referring physicians.

### Myocardial Perfusion Scintigraphy

MPS imaging at stress and rest was performed on the same day following international guidelines^18,19^ with a two-slice SPECT/CT (Symbia T2 TruePoint; Siemens Medical Solutions, USA) and intravenous injection of 250/750 MBq (stress/rest) of ^99m^Tc-tetrofosmin (Myoview; GE Company, Fairfield, CT, USA). Patients discontinued their cardiovascular medication overnight for stress application. Based on the individual physical fitness, the majority of patients (n = 199; 87%) underwent adenosine stress testing (Adenoscan©, Astellas Pharma US, Inc., Deerfield, IL, USA), and the remainder underwent bicycle ergometry with a target heart rate > 85% of maximal age-predicted heart rate (220-age). Gated SPECT/CT images with eight gates were acquired 30-60 minutes after injection with low-dose CT attenuation correction. Attenuation-corrected images were semi-quantitatively reconstructed and analyzed using Corridor4DM, version 6.1 (INVIA assisted by readers, Ann Arbor, University of Michigan Medical Centre) using an in-house camera and a sex-matched control group in a 17-segment. All segments were scored from 0 (no perfusion defect) to 4 (absence of tracer uptake). Scores were expressed as percentage of the maximum score (4 * 17=68) and perfusion defects were classified as minimal (1-4.9%), moderate (5-9.9%), and severe (≥ 10%).^13^ The summed rest score (SRS%) was used as an indicator of myocardial scarring, the summed stress score (SSS%) to assess scarring and reversible ischemia, and the summed difference score (SDS%) to quantify stress-induced ischemia. Clinical MPS analyses were reviewed by two experienced nuclear medicine physicians to verify or revise the original findings. The physicians were blinded to the DSE data but not to the clinical data and interpreted the MPS data by consensus.

### Dobutamine Stress Echocardiography

Transthoracic echocardiography (IE33, Philips Ultrasound, Bothell, USA, 1.3 to 4.2 MHz or VIVID7, GE Vingmed Ultrasound A/S, Horten, Norway, 2.2 to 5 MHz) was performed and evaluated by experienced echocardiographers according to international recommendations.^20–23^ During DSE, dobutamine was infused intravenously starting at 10 and then gradually increasing to 20, 30, and 40 μg·kg^−1^·min^−1^ every 2 minutes. If the target heart rate was not achieved, up to 1 mg atropine was given in doses of 0.25 mg. Regional wall motion abnormalities (WMAs) in the form of hypo- or akinesia were assessed visually. Dobutamine stress-induced WMA were an indicator of reversible ischemia, whereas fixed WMA were a marker for myocardial scarring. The interpretation of DSE in the clinical case report was reviewed for research purposes by an experienced cardiologist who was blinded to the MPS results but not the clinical data.

### Coronary Angiography (CA)

Pathologic findings in coronary arteries and their major branches were categorized by the extent of diameter stenosis as low-grade (< 50%), mid-grade (50%-70%), and high-grade (> 70%).^24^ Reversible ischemia in MPS or DSE was regionally assigned to the supplying artery/stenosis based on individual coronary anatomy. Because CA was part of the clinical workup, the cardiologists were not blinded to the previous diagnostics.

### Statistical Analysis

Baseline characteristics were compared using Fisher’s exact tests (categorical variables) or Mann-Whitney *U*-tests (continuous variables). Cohen’s κ was employed to monitor the inter-rater reliability of the two imaging modalities regarding fixed and reversible defects, and intraclass correlation coefficients quantify the agreement of left-ventricular measurements. Coronary events and survival after imaging were visualized by Kaplan–Meier curves, and distributions were compared with the log-rank test. Cox regression was used to estimate univariate hazard ratios (HRs) and adjusted HRs for relevant risk factors selected by stepwise backward elimination of non-significant variables at a level of *P* > .1. Statistical significance was defined as a *P*-value < .05 and confidence intervals (CIs) were set to 95%. Statistical analyses were performed using SPSS version 24 (SPSS, Inc. IBM, Armonk, USA).

## Results

Two-hundred twenty-nine ESRD patients without known CAD or typical angina pectoris were evaluated for KTx by MPS and DSE. Table 1 shows baseline characteristics and non-invasive imaging results at the time of screening. Median age was 51 years, the majority of patients were male (57.2%), and the median dialysis vintage was 19 months. Hyperlipidemia (98.3%) and hypertension (96.1%) were very common. Gated SPECT and echocardiography found left ventricular dilation and reduced ejection fractions in approximately every fourth ESRD patient and hypertrophic left ventricular myocardium in every second. Details of cardiac morphology and function are given in Supplementary Table 1.

**Table 1:** Baseline characteristics categorized by findings of myocardial perfusion scintigraphy (MPS) and dobutamine stress echocardiography (DSE)

Median (25% quartile, 75% quartile) or n [%]					
	Normal MPS (n = 193)	Abnormal MPS (n = 36)	Normal DSE (n = 192)	Abnormal DSE (n = 37)	Total (n = 229)
Age (years)	51 (42, 60)	51 (46, 64)	50 (42, 60)	54 (46, 64)	51 (42, 61)
Gender (male)	106 [54.9%]	25 [69.4%]	102 [53.1%]*	29 [78.4%]*	131 [57.2%]
BMI (kg·m^−2^)	26.0 (22.5, 29.9)	25.2 (22.5, 28.1)	25.8 (22.5, 30.1)	25.7 (22.5, 28.1)	25.8 (22.5, 29.3)
Diabetes mellitus	33 [17.1%]	5 [13.9%]	31 [16.1%]	7 [18.9%]	38 [16.6%]
Current smoker	36 [18.7%]	10 [27.8%]	38 [19.8%]	8 [21.6%]	46 [20.1%]
Duration of dialysis (months)	16 (6, 35)*	27 (17, 56)*	18 (7, 36)	22 (9, 57)	19 (7, 37)
Hyperlipidemia	189 [97.9%]	36 [100%]	190 [99.0%]	35 [94.6%]	225 [98.3%]
LDL-cholesterol (mg·dL^−1^)	105 (85, 137)	108 (92, 151)	105 (85, 139)	108 (92, 126)	106 (85, 137)
Hypertension	184 [95.3%]	36 [100%]	183 [95.3%]	37 [100%]	220 [96.1%]
Mean blood pressure (mmHg)	107 (97, 117)	107 (97, 117)	107 (97, 117)	107 (97, 117)	107 (97, 117)
Previous stroke	8 [4.1%]	3 [8.3%]	4 [2.1%]*	7 [18.9%]*	11 [4.8%]
Family history of MI or stroke	43 [22.3%]	7 [19.4%]	42 [21.9%]	8 [21.6%]	50 [21.8%]
LV-dilatation	41 [21.2%]	13 [36.1%]	42 [21.9%]	12 [32.4%]	54 [23.6%]
LV-hypertrophy	97 [50.3%]	19 [52.8%]	98 [51.0%]	18 [48.6%]	116 [50.7%]
Reduced LV-ejection fraction	48 [24.9%]*	18 [50.0%]*	46 [24.0%]*	20 [54.1%]*	66 [28.8%]

Myocardial perfusion scintigraphy (MPS) was assigned abnormal in case of any perfusion defects and dobutamine stress echocardiography (DSE) was assigned abnormal in case of any wall motion abnormalities

Values are tested for differences between groups of normal and abnormal imaging result by Mann-Whitney-*U*-test or by Fisher’s exact test: *P-value < .05

### Myocardial Perfusion and Contractile Reserve

#### MPS

Overall, 36 of the 229 patients (15.7%) exhibited abnormal stress perfusion (SSS% ≥ 1) in MPS. Fixed perfusion defects at rest (SRS% ≥ 1), indicative of myocardial scarring, were detected in 13 patients (5.7%). Reversible ischemia (SDS% ≥ 1) occurred in 31 patients (13.5%), of whom 8 patients (3.5%) had perfusion defects already at rest. Patients with impaired myocardial perfusion were on dialysis significantly longer and more often had reduced left ventricular ejection fraction compared to patients with normal MPS.

#### DSE

DSE revealed local WMAs at rest and/or during stress in 37 of 229 patients (16.2%). Hypo- or akinesia at rest as a sign of myocardial scarring was observed in 18 ESRD patients (7.9%). Regional strain-induced worsening of myocardial contractility, indicative for reversible ischemia, was noted in 28 patients (12.2%), of whom 9 patients (3.9%) already had WMAs at rest. Compared to patients with normal DSE, those with WMAs were more often male and had reduced left ventricular ejection fraction.

### Head-to-Head Comparison of MPS and DSE

#### Fixed perfusion defects vs WMAs

In 203 of 229 ESRD patients (88.6%), both MPS and DSE were normal at rest, whereas both found myocardial scarring in 5 patients (2.2%), of which the exact localization differed between the two modalities in one patient. However, in a total of 21 ESRD patients (9.2%), MPS and DSE at rest showed contradictory results (Cohen’s κ 0.275; *P* < .001) (Table 2).

**Table 2:** Agreement between fixed perfusion defects in myocardial perfusion scintigraphy (MPS) and fixed wall motion abnormalities in dobutamine stress echocardiography (DSE)

Fixed defects	DSE−	DSE+	Total
MPS−	203 (88.6%)	13 (5.7%)	216 (94.3%)
MPS+	8 (3.5%)	5 (2.2%)	13 (5.7%)
Total	211 (92.1%)	18 (7.9%)	229 (100%)

Cohen‘s κ = 0.275 (*P* < .001)

“MPS+” represents perfusion defects at rest; “DSE+” represents wall motion abnormalities at rest

#### Stress-inducible perfusion defects vs WMAs

As in resting studies, the results of MPS and DSE showed a good overall agreement in stress studies in ESRD patients. In 183 patients (79.9%), myocardial ischemia was not present in either MPS or DSE. Another 13 patients (5.7%) had ischemia signs in both MPS and DSE, and in 10 of the 13 patients, the exact localization matched. MPS and DSE disagreed in 33 cases (14.4%) (Cohen’s κ 0.358; *P* < .001) (Table 3).

**Table 3:** Agreement between reversible perfusion defects in myocardial perfusion scintigraphy (MPS) and reversible wall motion abnormalities in dobutamine stress echocardiography (DSE)

Reversible defects	DSE−	DSE+	Total
MPS−	183 (79.9%)	15 (6.6%)	198 (86.5%)
MPS+	18 (7.9%)	13 (5.7%)	31 (13.5%)
Total	201 (87.8%)	28 (12.2%)	229 (100%)

Cohen’s κ = 0.358 (*P* < .001)

“MPS+” represents stress-induced perfusion defects; “DSE+” represents stress-induced wall motion abnormalities

In the adenosine MPS group, MPS and DSE matched in 88.2% and in the bicycle MPS group, MPS and DSE matched in 80.6% regarding reversible defects. Due to the small size of the bicycle group (31 patients), the difference was not significant (*P* = .152).

#### Cardiac function

Functional measurements from gated SPECT and echocardiography showed good agreements in LVEDV and LVESV [intraclass correlation (ICC) = 0.794 and 0.810, respectively] and LVEF (ICC = 0.612) (Supplementary Figure 1).

### Coronary Angiography

Of 46 patients with stress-related ischemia in MPS and/or DSE, 38 (82.6%) were ultimately referred for CA on clinical grounds. Results of CA for patients with signs of ischemia in DSE and/or MPS are presented in Table 4. Of the 15 patients with a stenosis ≥ 50%, 11 (73.3%) had ischemia in MPS and 8 (53.3%) had ischemia in DSE. In these 15 patients, matching localizations of the stenoses were found in 10 of 11 patients (90.9%) regarding ischemia in MPS and in 7 of 8 patients (87.5%) regarding ischemia in DSE. A higher SDS% cut-off score in MPS did not increase the incidence of stenoses ≥ 50% by angiography.

**Table 4:** Results of coronary angiography in clinically selected patients with stress-induced ischemia in myocardial perfusion scintigraphy and/or dobutamine stress echocardiography (n = 38/229)

No stenosis	9 (23.7%)
Low-grade stenosis	14 (36.8%)
Mid-grade stenosis	5 (13.2%)
High-grade stenosis	10 (26.3%)

Stenoses are classified by their extent of diameter narrowing in low-grade (< 50%), mid-grade (50%-70%) and high-grade (> 70%)

### Follow-up

**Figure 1:**
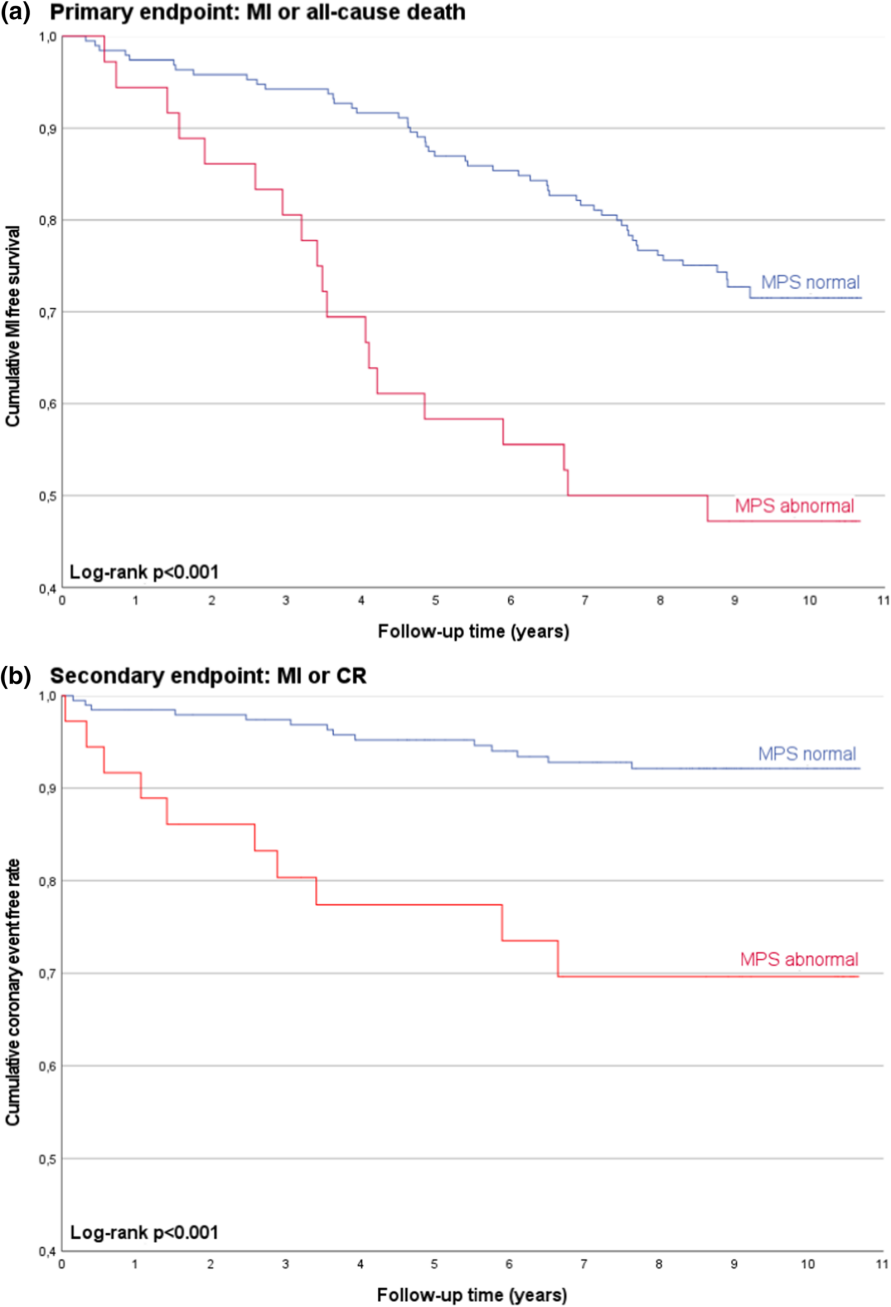
Clinical outcomes stratified for perfusion defects in MPS. The figure shows Kaplan-Meier survival plots among patients with and without perfusion defects in MPS (MPS abnormal/normal) for the primary endpoint (composite of MI and all-cause death) (**A**) and for the secondary endpoint (composite of MI and coronary revascularization) (**B**). Distributions were compared by the log-rank test. *MI*, myocardial infarction; *CR*, coronary revascularization; *MPS*, myocardial perfusion scintigraphy

**Figure 2:**
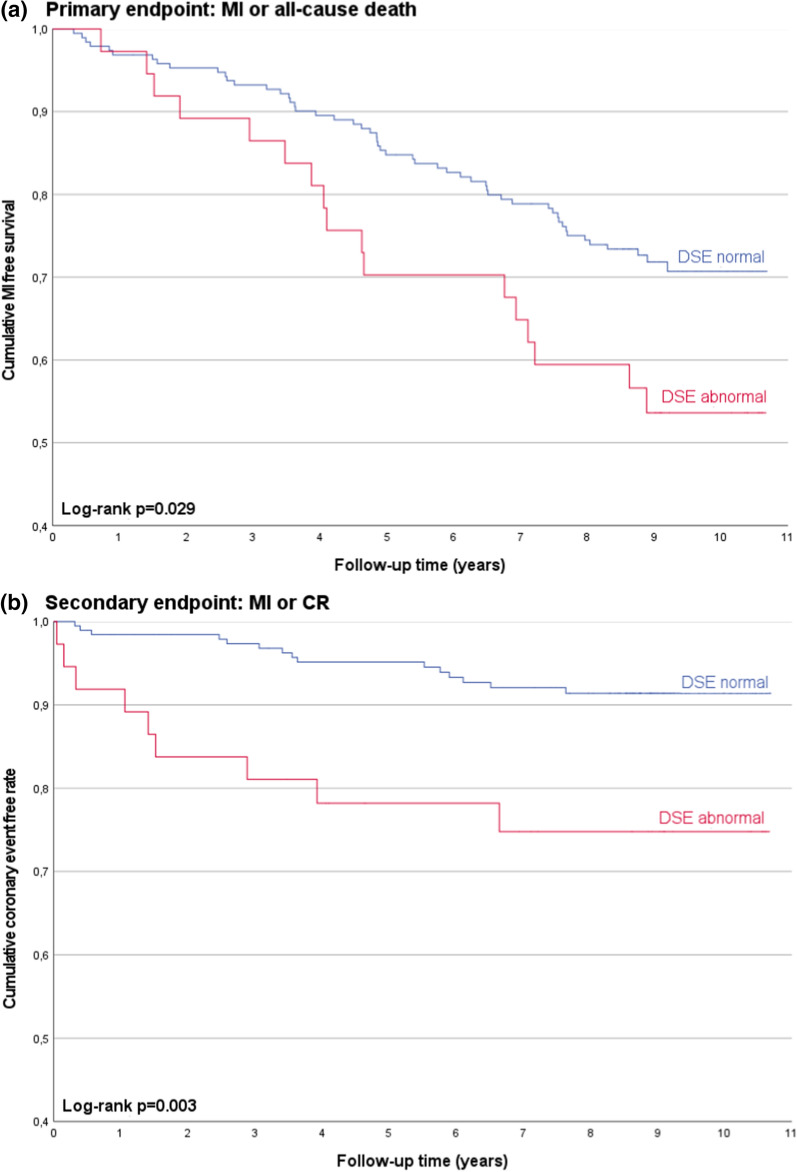
Clinical outcomes stratified for wall motion abnormalities in DSE. Depicted are Kaplan-Meier survival plots stratified by the presence or absence of wall motion abnormalities (DSE abnormal/normal) in DSE for the primary endpoint (composite of MI and all-cause death) (**A**) and for the secondary endpoint (composite of MI and coronary revascularization) (**B**). Distributions were compared by the log-rank test. *MI*, myocardial infarction; *CR*, coronary revascularization; *DSE*, dobutamine stress echocardiography

Median follow-up time for cardiovascular events and death was 8 years and 10 months. During this period, 146 of the 229 patients (63.8%) underwent KTx and 8 patients (3.5%) had discontinued follow-up in the interim. Acute MI and stroke occurred in 17 patients each, and 61 patients died. The most common causes of death were infectious (20 patients, 32.8%), cardiovascular (11 patients, 18%), and cancer-related (7 patients, 11.5%). In 16 patients, the cause of death was unknown or uncertain. Coronary revascularisation was performed in 18 patients (7.9%) by PCI and in 11 patients (4.8%) by CABG. The primary endpoint included MI or all-cause death and occurred in 70 patients (30.6%). The secondary endpoint, which was defined as a composite of MI or CR not triggered by baseline MPS or DSE, was observed in 24 patients (10.5%). Results of the univariate Cox regression and unadjusted HRs are given in Table 5. Age and dialysis vintage were the strongest predictors for adverse events, followed by perfusion defects in MPS (abnormal MPS) and WMA in DSE (abnormal DSE). Additionally, a reduced LV-ejection fraction was a significant risk factor for the primary but not for the secondary endpoint. Figures 1 and 2 illustrate the corresponding Kaplan-Meier curves stratified by normal or abnormal MPS or DSE, respectively. Multivariate Cox regression was performed by stepwise eliminating irrelevant variables over a level of *P* > .1 in separate models including either MPS (model a) or DSE (model b). Remaining risk factors were only age, dialysis vintage, and perfusion defects in MPS (model a) and WMA in DSE (model b). After adjustment to the selected variables, perfusion defects in MPS were still a significant prognostic risk factor for the primary (adjusted HR 1.77; 95% CI 1.02-3.08; *P* = .043) and secondary endpoint (adjusted HR 3.21; 95% CI 1.35-7.61; *P* = .008) (Table 6, model a). WMA in DSE remained significant after multivariate adjustment only for the secondary endpoint (adjusted HR 2.67; 95% CI 1.15-6.20; *P* = .022) (Table 6, model b). Combining the two imaging modalities only modestly increased prognostic significance. In patients with abnormal MPS and/or abnormal DSE, the adjusted HRs were 1.92 (95% CI 1.17-3.17; *P* = .01) for the primary and 3.45 (95% CI 1.49-8.01; *P* = .04) for the secondary endpoint.

**Table 5:** Unadjusted Cox regression for primary and secondary endpoint

	MI or all-cause death (primary endpoint)		MI or CR (secondary endpoint)	
	HR (95% CI)	*P*-value	HR (95% CI)	*P*-value
Perfusion defect in MPS	2.57 (1.51-4.36)	< .001	4.70 (2.08-10.59)	< .001
WMA in DSE	1.84 (1.06-3.18)	.029	3.57 (1.56-8.17)	.003
Age (years)	1.06 (1.04-1.08)	< .001	1.05 (1.01-1.09)	.009
Gender (male)	1.38 (0.85-2.25)	.197	1.54 (0.66-3.59)	.320
BMI (kg·m^−2^)	1.02 (0.97-1.07)	.420	0.98 (0.90-1.07)	.656
Diabetes mellitus	1.70 (0.97-2.96)	.064	1.43 (0.53-3.82)	.480
Current smoker	1.00 (0.56-1.80)	.991	2.01 (0.86-4.69)	.107
Duration of dialysis (months)	1.01 (1.01-1.02)	.001	1.01 (1.01-1.02)	.002
Hyperlipidemia	1.15 (0.16-8.27)	.891	0.39 (0.05-2.89)	.356
LDL-cholesterol (mg·dL^−1^)	1.01 (1.00-1.01)	.152	1.01 (0.99-1.02)	.296
Hypertension	3.17 (0.44-22.81)	.252	21.48 (0.01-134.98)	.492
Mean blood pressure (mmHg)	1.00 (0.98-1.01)	.656	1.01 (0.98-1.04)	.560
Previous stroke	1.45 (0.58-3.60)	.424	0.79 (0.11-5.82)	.813
Family history of MI or stroke	1.45 (0.86-2.44)	.161	0.50 (0.15-1.69)	.266
LV-dilatation	1.23 (0.73-2.08)	.444	1.66 (0.71-3.88)	.242
LV-hypertrophy	1.20 (0.75-1.93)	.446	0.57 (0.25-1.29)	.175
Reduced LV-ejection fraction	1.78 (1.10-2.87)	.019	1.29 (0.55-3.01)	.557
KTx during follow-up (time-dependent)	0.64 (0.39-1.06)	.086	0.40 (0.14-1.15)	.091

*MI*, myocardial infarction; *CR*, coronary revascularization; *MPS*, myocardial perfusion scintigraphy; *WMA*, wall motion abnormality; *DSE*, dobutamine stress echocardiography; *HR*, unadjusted hazard ratio; *KTx*, kidney transplantation

**Table 6:** Adjusted Cox regression for primary and secondary endpoint including either MPS (model a) or DSE (model b) Covariates were selected by stepwise backward elimination depending on the likelihood-ratio

	MI or all-cause death (primary endpoint)		MI or CR (secondary endpoint)	
	HR (95% CI)	*P*-value	HR (95% CI)	*P*-value
a				
Perfusion defect in MPS	1.77 (1.02-3.08)	.043	3.21 (1.35-7.61)	.008
Age (years)	1.06 (1.03-1.08)	< .001	1.05 (1.01-1.08)	.019
Duration of dialysis (months)	1.01 (1.01-1.02)	.002	1.01 (1.00-1.02)	.012
b				
WMA in DSE	1.36 (0.78-2.37)	.279	2.67 (1.15-6.20)	.022
Age (years)	1.06 (1.04-1.08)	< .001	1.05 (1.01-1.09)	.014
Duration of dialysis (months)	1.01 (1.01-1.02)	< .001	1.02 (1.01-1.02)	.002

*MPS*, myocardial perfusion scintigraphy; *WMA*, wall motion abnormality; *DSE*, dobutamine stress echocardiography; *MI*, myocardial infarction; *CR*, coronary revascularization; *HR*, adjusted hazard ratio

Stenosis > 50% in CA was an additional predictor of adverse cardiac events. MI was observed in 4 of 15 patients (26.7%) with stenosis > 50% vs only one of 23 patients (4.3%) in the group without relevant coronary stenosis. Because of the low rate of CA in the patients studied, no statistically significant conclusion can be drawn.

Decision to KTx was grossly independent of results of pathologic stress testing or angiography. Patients with pathological stress testing underwent KTx equally often as patients with normal stress testing (71.2% vs 61.6%, *P* > .05). In the first month after transplantation, one patient died because of MI and two patients had non-fatal MIs. There were no periprocedural strokes or lethal infections. In patients who underwent KTx during the follow-up period, the primary endpoint occurred in 21.9% (32/146) compared to 54.2% (45/83) in patients remaining on dialysis, the secondary endpoint occurred in 8.9% (13/146) vs 13.3% (11/83). However, this trend was not statistically significant as KTx as a time-dependent covariate in a univariate and adjusted Cox regression analysis had no effect on the primary (HR 0.64; 95% CI 0.39-1.06; *P* = .086) or the secondary endpoint (HR 0.40; 95% CI 0.14-1.15; *P* = .091).

## Discussion

This study found high cardiovascular morbidity and all-cause mortality in a clinically well-characterized cohort of 229 adult ESRD patients without known CAD or typical angina pectoris at baseline. Long-term follow-up showed that myocardial perfusion defects in stress MPS were a strong predictor of the composite primary endpoint (MI or all-cause death) and the secondary endpoint (MI or CR). WMAs in DSE were also found to be prognostically significant, particularly with respect to the secondary, coronary endpoint. Analysis of traditional and non-traditional risk factors highlights advanced age and dialysis vintage as crucial parameters for adverse coronary events and mortality in these patients. Although KTx during the follow-up period showed a trend towards a better outcome, this was not statistically significant according to time-dependent Cox regression analysis.

The findings of MPS and DSE in this study and their prognostic significance are comparable to previous studies that evaluated one of the two imaging modalities but not both in the same patient.^13,15,16,25,26^ To our knowledge, this is the first study to directly compare the prognostic value of the two most commonly used cardiac imaging tools in ESRD patients.

The two imaging modalities matched only in 196 out of 229 patients (85.6%; κ = 0.358) regarding reversible defects and in 208 out of the 229 patients (90.8%; κ = 0.275) regarding fixed defects. This can only be compared to a single previous study evaluating 49 ESRD patients,^27^ which found an agreement on the presence or absence of reversible defects between MPS and DSE of 69% (κ = 0.25), which is even lower than in the present study. Discrepancies between findings of MPS and DSE in individual patients could be attributed to the different (patho-) physiological parameters measured by the two imaging modalities. MPS measures perfusion at rest and during hyperaemia (vasodilation by adenosine in the present study), hence myocardial perfusion reserve. This is a sensitive measure of the overall functionality of the cardiac arterial vasculature, integrating all its components from epicardial arteries to arterioles and capillaries. In comparison, DSE assesses regional contractility at baseline and under dobutamine, hence contractile reserve. It is therefore not a direct but indirect measure of perfusion reserve, which can only be detected at a later stage of the ischemia cascade.^28^ Secondly, disagreements may be triggered by the different stress protocols of the two imaging modalities. Vasodilatation upon adenosine is partly impaired in conditions of endothelial dysfunction such as diabetes,^29^ which can influence the results of DSE. Inaccuracies could also partly be attributed to failure to achieve target heart rate.^30^ Nevertheless, it suggests that MPS and DSE provide complementary prognostic information, because abnormal MPS and/or DSE were more strongly correlated with adverse events compared with only a single abnormal imaging result.

An interesting finding of our study is that in patients with reversible ischemia detected by MPS and/or DSE who were subsequently referred to CA, the positive predictive value (PPV) for a coronary stenosis of more than 50% was only 44% in MPS and only 31% in DSE. This is discrepant to a review of Parikh et al. who found a pooled PPV for an obstructive CAD of 70% for MPS and of 76% for DSE.^31^ Possible explanations are differing inclusion criteria for patients with previous CAD who are known to have higher agreements between MPS/DSE and angiography as well as higher cut-off scores in the reviewed MPS studies. However, in this study, higher cut-off scores for MPS did not increase the PPV for stenoses ≥ 50%. This is consistent with the finding of Helve et al. that even mild perfusion defects in MPS proved to have a high prognostic impact on cardiovascular mortality in ESRD patients before KTx.^13^ This again contrasts with patients with preserved kidney function and CAD.^13^ Likewise, Doukky et al showed that ESRD patients with a summed stress score < 4 in MPS are at high risk for cardiovascular events.^25^ This is another argument to lower the cut-off score for pathologic results in ESRD patients, as was done in this study.

Negative angiograms in presence of ischemia on imaging could possibly be explained by the pathophysiological nature of ESRD-associated vasculopathies in contrast to classical CAD. While CAD results in epicardial stenoses in the majority of cases, uremic cardiovasculopathy manifests with a higher frequency of microvasculopathy, impairment of endothelial function, and myocardial fibrosis.^32^ Interestingly, in a subanalysis of this study, patients with ischemia in MPS and/or DSE but without relevant stenosis > 50% in CA were not at high risk for future MI. On the other hand, considering the therapeutic utility of CA, patients with ischemia and a relevant coronary stenosis were still at high risk for coronary adverse events despite of receiving CR. The efficacy of CA in ESRD patients is also criticized in a recent state-of-the-art review^33^ and was further investigated by Bangalore et al, who found a high incidence of MI and all-cause death in patients with advanced CKD and no benefit of an invasive compared with a conservative strategy.^34^

Patients with ESRD are at high risk to develop coronary vasculopathy due to conventional and kidney-related risk factors, which seriously impairs their prognosis.^3,5^ In our study cohort of ESRD patients, traditional risk factors for CAD were frequently present but had little impact on non-invasive imaging outcome and long-term prognosis, with the exception of patient age. This is consistent with studies showing low accuracy of traditional risk scores on the outcome of ESRD patients.^35^ However, studies including patients with prior CAD—in contrast to our study—found smoking and diabetes to be independent predictors for cardiovascular events in ESRD patients.^36,37^ It might also be a promising approach to survey at the accumulated burden of risk factors including traditional risk factors as well as left-ventricular hypertrophy and dialysis vintage as defined by the AHA/ACC statement.^11,38^

In our patient cohort, left ventricular hypertrophy was frequently detected, whereas left ventricular volumes and ejection fraction were normal on average. These findings are consistent with literature on cardiac magnetic resonance imaging regarding so-called uremic cardiomyopathy.^39^

### Limitations

Thresholds for summed stress scores for MPS are defined for CAD diagnostics but are not yet validated in ESRD patients due to differences in the underlying vascular pathology and remain unclear up to date. Due to the clinical context of this study, data on CA is limited because it was performed only in selected patients and the angiographers were not blinded to the results of non-invasive imaging. Furthermore, routine CA did not include measurements of fractional flow reserve or microcirculatory resistance as functional parameters that could have improved its value.

### Conclusion

Perfusion defects in MPS and WMAs in DSE are frequently present in ESRD patients without known CAD and can predict coronary adverse events. Compared with DSE, abnormal MPS, even with small perfusion deficits, is a stronger predictor of all-cause mortality, MI, and the need for future CR. The functional information obtained by MPS and DSE is complementary but to some extent contradictory, which may indicate differences in the underlying pathophysiology.

## New Knowledge Gained

- MPS and DSE reveal a significant incidence of ischemia and myocardial scarring in kidney transplant candidates without known CAD.
- Due to the different pathophysiological parameters measured, MPS and DSE provide complementary but sometimes contradictory information in a significant number of patients.
- Perfusion defects in MPS have a higher prognostic significance for all-cause mortality, MI, and the need for future CR, compared with WMAs in DSE.

## Supplementary Information

Below is the link to the electronic supplementary material.

Supplementary file1 (DOCX 192 kb)

## Abbreviations

MPS: Myocardial perfusion scintigraphy
DSE: Dobutamine stress echocardiography
CA: Coronary angiography
CKD: Chronic kidney disease
ESRD: End-stage renal disease
KTx: Kidney transplantation
WMA: Wall motion abnormalities
GFR: Glomerular filtration rate
CR: Coronary revascularization
MI: Myocardial infarction

## References

[1] Levey A, Atkins R, Coresh J (2007). Chronic kidney disease as a global public health problem: Approaches and initiatives—A position statement from Kidney Disease Improving Global Outcomes. Kidney Int.

[2] Myers OB, Pankratz VS, Norris KC (2018). Surveillance of CKD epidemiology in the US—A joint analysis of NHANES and KEEP. Sci Rep.

[3] Matsushita K, van der Velde M, Astor BC (2010). Association of estimated glomerular filtration rate and albuminuria with all-cause and cardiovascular mortality in general population cohorts: A collaborative meta-analysis. Lancet.

[4] Edwards NC, Moody WE, Chue CD (2014). Defining the natural history of uremic cardiomyopathy in chronic kidney disease: The role of cardiovascular magnetic resonance. JACC Cardiovasc Imaging.

[5] Go AS, Chertow GM, Fan D (2004). Chronic kidney disease and the risks of death, cardiovascular events, and hospitalization. N Engl J Med.

[6] Gansevoort RT, Correa-Rotter R, Hemmelgarn BR (2013). Chronic kidney disease and cardiovascular risk: Epidemiology, mechanisms, and prevention. Lancet.

[7] Vervloet M, Cozzolino M (2017). Vascular calcification in chronic kidney disease: Different bricks in the wall?. Kidney Int.

[8] Tumlin JA, Costanzo MR, Chawla LS (2013). Cardiorenal syndrome type 4: Insights on clinical presentation and pathophysiology from the eleventh consensus conference of the Acute Dialysis Quality Initiative (ADQI). Contrib Nephrol.

[9] Di Marco GS, Reuter S, Kentrup D (2011). Cardioprotective effect of calcineurin inhibition in an animal model of renal disease. Eur Heart J.

[10] Piepoli MF, Hoes AW, Agewall S (2016). 2016 European Guidelines on cardiovascular disease prevention in clinical practice. Eur Heart J.

[11] Lentine KL, Costa SP, Weir MR (2012). Cardiac disease evaluation and management among kidney and liver transplantation candidates. JAC.

[12] K/DOQI Workgroup (2005). K/DOQI clinical practice guidelines for cardiovascular disease in dialysis patients. Am J Kidney Dis.

[13] Rabbat CG (2003). Prognostic value of myocardial perfusion studies in patients with end-stage renal disease assessed for kidney or kidney–pancreas transplantation: A meta-analysis. J Am Soc Nephrol.

[14] Helve S, Laine M, Sinisalo J (2017). Even mild reversible myocardial perfusion defects predict mortality in patients evaluated for kidney transplantation. Eur Heart J Cardiovasc Imaging.

[15] Kim J-K, Kim SG, Kim HJ, Song YR (2012). Cardiac risk assessment by gated single-photon emission computed tomography in asymptomatic end-stage renal disease patients at the start of dialysis. J Nucl Cardiol.

[16] Bergeron S, Hillis GS, Haugen EN (2007). Prognostic value of dobutamine stress echocardiography in patients with chronic kidney disease. Am Heart J.

[17] Wang LW, Fahim MA, Hayen A (2011). Cardiac testing for coronary artery disease in potential kidney transplant recipients. Cochrane Database Syst Rev.

[18] Verberne HJ, Acampa W, Anagnostopoulos C (2015). EANM procedural guidelines for radionuclide myocardial perfusion imaging with SPECT and SPECT/CT. Eur J Nucl Med Mol Imaging.

[19] Henzlova MJ, Duvall WL, Einstein AJ (2016). ASNC imaging guidelines for SPECT nuclear cardiology procedures: Stress, protocols, and tracers. J Nucl Cardiol.

[20] Marwick TH, Gillebert TC, Aurigemma G (2015). Recommendations on the use of echocardiography in adult hypertension: A report from the European Association of Cardiovascular Imaging (EACVI) and the American Society of Echocardiography (ASE). Eur Heart J Cardiovasc Imaging.

[21] Lang RM, Badano LP, Mor-Avi V (2015). Recommendations for cardiac chamber quantification by echocardiography in adults: An update from the American Society of Echocardiography and the European Association of Cardiovascular Imaging. J Am Soc Echocardiogr.

[22] Sicari R, Nihoyannopoulos P, Evangelista A (2008). Stress echocardiography expert consensus statement. Eur J Echocardiogr.

[23] Pellikka PA, Nagueh SF, Elhendy AA (2007). American Society of Echocardiography recommendations for performance, interpretation, and application of stress echocardiography. J Am Soc Echocardiogr.

[24] Patel MR, Steven Bailey C-CR, Robert Bonow C-CO (2012). ACCF/SCAI/AATS/AHA/ASE/ASNC/HFSA/HRS/SCCM/SCCT/SCMR/STS 2012 appropriate use criteria for diagnostic catheterization. JAC.

[25] Doukky R, Fughhi I, Campagnoli T (2017). The prognostic value of regadenoson SPECT myocardial perfusion imaging in patients with end-stage renal disease. J Nucl Cardiol.

[26] Wang LW, Masson P, Turner RM (2015). Prognostic value of cardiac tests in potential kidney transplant recipients: A systematic review. Transplantation.

[27] Bart BA, Cen YY, Hendel RC (2009). Comparison of dobutamine stress echocardiography, dobutamine SPECT, and adenosine SPECT myocardial perfusion imaging in patients with end-stage renal disease. J Nucl Cardiol.

[28] Stillman AE, Oudkerk M, Bluemke DA (2018). Imaging the myocardial ischemic cascade. Int J Cardiovasc Imaging.

[29] Ragosta M, Samady H, Isaacs RB (2004). Coronary flow reserve abnormalities in patients with diabetes mellitus who have end-stage renal disease and normal epicardial coronary arteries. Am Heart J.

[30] Palepu S, Prasad R (2015). Screening for cardiovascular disease before kidney transplantation. World J Transplant.

[31] Parikh K, Appis A, Doukky R (2015). Cardiac imaging for the assessment of patients being evaluated for kidney or liver transplantation. J Nucl Cardiol.

[32] Radhakrishnan A, Pickup LC, Price AM (2019). Coronary microvascular dysfunction: A key step in the development of uraemic cardiomyopathy?. Heart J.

[33] Sarnak MJ, Amann K, Bangalore S (2019). Chronic kidney disease and coronary artery disease: JACC state-of-the-art review. J Am Coll Cardiol.

[34] Bangalore S, Maron DJ, O’Brien SM (2020). Management of coronary disease in patients with advanced kidney disease. N Engl J Med.

[35] Weiner DE, Tighiouart H, Elsayed EF (2007). The Framingham predictive instrument in chronic kidney disease. J Am Coll Cardiol.

[36] Furuhashi T, Moroi M, Awaya T (2014). Usefulness of stress myocardial perfusion imaging and baseline clinical factors for predicting cardiovascular events in patients with prior coronary artery disease. Circ J.

[37] Shlipak MG, Fried LF, Cushman M (2005). Cardiovascular mortality risk in chronic kidney disease: Comparison of traditional and novel risk factors. JAMA.

[38] Doukky R, Fughhi I, Campagnoli T (2018). Validation of a clinical pathway to assess asymptomatic renal transplant candidates using myocardial perfusion imaging. J Nucl Cardiol.

[39] Mark PB, Johnston N, Groenning BA (2006). Redefinition of uremic cardiomyopathy by contrast-enhanced cardiac magnetic resonance imaging. Kidney Int.

