# Supplementary material for: Prognostic implication of myocardial perfusion and contractile reserve in end-stage renal disease: A direct comparison of myocardial perfusion scintigraphy and dobutamine stress echocardiography
Source: J Nucl Cardiol. 2021 Nov 8;29(6):2988–99. doi: 10.1007/s12350-021-02844-y (PMC9834353; doi:10.1007/s12350-021-02844-y)
Supplement: Supplementary file 1 — Supplementary file1 (DOCX 192 kb) [file 12350_2021_2844_MOESM1_ESM.docx]

**Prognostic implication of myocardial perfusion and contractile reserve in end-stage renal disease: A direct comparison of myocardial perfusion scintigraphy and dobutamine stress echocardiography**

Joachim Bautz^1,3^, Jörg Stypmann^2^, Stefanie Reiermann^3^, Hermann-Joseph Pavenstädt^3^, Barbara Suwelack^3^, Lars Stegger^1^, Kambiz Rahbar^1^, Stefan Reuter^3*^ and Michael Schäfers^1,4,5*^

1. Department of Nuclear Medicine, University Hospital Münster, Germany
2. Department of Cardiology, University Hospital Münster, Germany
3. Department of Internal Medicine D, Nephrology, University Hospital Münster, Germany
4. European Institute for Molecular Imaging, University of Münster, Germany
5. DFG EXC 1003 ‘Cells in Motion’ Cluster of Excellence, University of Münster, Germany
   *equal contribution

Corresponding author:
Michael SCHÄFERS, Department of Nuclear Medicine, University Hospital Münster,
Albert-Schweitzer-Campus 1, Building A1, 48149, Münster, Germany.
E-mail: schafmi@uni-muenster.de
Tel.: +49 251/83-47362

Acknowledgments:
The authors would like to thank the technicians at the Department of Nuclear Medicine of the University Hospital Münster and Raphael Koch and Dennis Görlich (Institute of Biometry and Clinical Research, University of Münster) for statistical support.

Funding and disclosure:
This study was supported in part by the Sonderforschungsbereich SFB 656, Molecular Cardiovascular Imaging; Münster, Germany (projects C2 and C6) and by an institutional grant of Siemens Medical Solutions (Erlangen, Germany). Joachim Bautz, Jörg Stypmann, Stefanie Reiermann, Hermann-Joseph Pavenstädt, Barbara Suwelack, Lars Stegger, Kambiz Rahbar, Stefan Reuter and Michael Schäfers declare that they have no conflict of interest.

**Supplemental data**

| **Supplemental table 1** Functional parameters assessed by gated SPECT and echocardiography (n=229)  Median (25% quartile, 75% quartile) [Reference values [20,21]] | | | | |
| --- | --- | --- | --- | --- |
|  | Gated SPECT | | Echocardiography | |
|  | Men (n=131) | Women (n=98) | Men (n=131) | Women (n=98) |
| LVEDV [ml] | 120 (93,155) [<165] | 90 (76,116) [<103] | 114 (88,142) [62-150] | 89 (76,117) [46-106] |
| LVESV [ml] | 46 (30,64) [<64] | 29 (21,38) [<33] | 43 (34,57) [21-61] | 33 (24,42) [14-42] |
| LVPW [cm] | - | - | 1.2 (1.0,1.2) [0.6-1.0] | 1.0 (0.9,1.2) [0.6-0.9] |
| IVS [cm] | - | - | 1.3 (1.1,1.4) [0.6-1.0] | 1.1 (1.0,1.3) [0.6-0.9] |
| LVEF [%] | 61 (55,68) [>58] | 70 (64,74) [>63] | 63 (59,66) [52-72] | 64.5 (60,68) [54-74] |
| *LVEDV* left ventricular end-diastolic volume, *LVESV* left ventricular end-systolic volume, *LVPW* left ventricular posterior wall, *IVS* intraventricular septum, *RWT* relative wall thickness, *LVEF* left ventricular ejection fraction. | | | | |

**
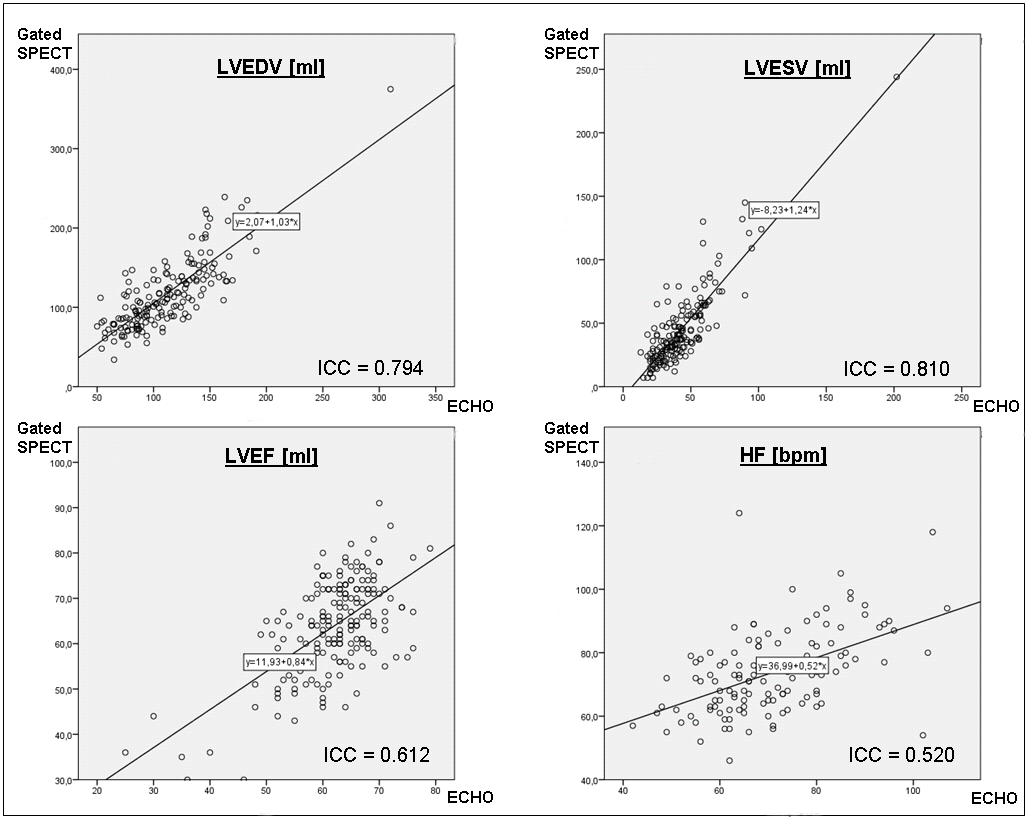
**

**Suppl. fig. 1** Morphological and functional measurements from gated SPECT and echocardiography of the 229 patients are visualized by scatter plots. Intraclass correlation coefficients show a high agreement in volume measurements and a moderate agreement in left ventricular systolic function. *LVEDV* left ventricular end-diastolic volume, *LVESV* left ventricular end-systolic volume, *LVEF* left ventricular ejection fraction, *HF* heart rate.
